# Lack of Galectin-3 Disturbs Gut–Adipose–Liver Axis in High-Fat-Diet Mice Model

**DOI:** 10.3390/biomedicines14061288

**Published:** 2026-06-05

**Authors:** Flávia R. S. Corrêa, Natália G. Mação, Felipe S. Lemos, Victor F. S. Ferreira, Vinícius F. Carvalho, Felipe L. Oliveira

**Affiliations:** 1 Institute of Biomedical Sciences, Federal University of Rio de Janeiro, Rio de Janeiro 21941-901, Brazil; flaviareginasobreira@gmail.com (F.R.S.C.); nataliamacao@gmail.com (N.G.M.); freire.victor@yahoo.com.br (V.F.S.F.); 2 Laboratory of Cellular and Molecular Neurobiology, Carlos Chagas Filho Biophysics Institute, Federal University of Rio de Janeiro, Rio de Janeiro 21941-901, Brazil; simoeslemos.f@gmail.com; 3 National Institute of Science and Technology in Neuroimmunomodulation (INCT.NIM), Laboratory of Inflammation, Oswaldo Cruz Institute, Oswaldo Cruz Foundation, Rio de Janeiro 21040-360, Brazil; vinicius.frias@fiocruz.br

**Keywords:** gut–liver axis, high-fat diet, galectin-3, obesity, MASLD

## Abstract

**Background/Objectives**: A high-fat diet (HFD) promotes hepatic steatosis, inflammation, and systemic metabolic imbalance. Notably, HFDs can affect the gut–liver axis and adipose tissue homeostasis. Galectin-3 (Gal-3) binds to β-galactosides and plays regulatory roles in the gut–liver axis, connecting metabolic stress with inflammation and tissue remodelling. The objective of this study was to investigate whether Gal-3 affects the gut–liver axis and adipose tissue biology after HFD supplementation. **Methods**: Six-week-old C57BL/6 mice were randomly divided into either wild-type (*Lgals3^+/+^*) or knockout (*Lgals3^−/−^*) groups. Both groups received an HFD orally for 12 weeks, along with their respective control groups. Physiological measurements and microscopic examination of the gut, liver, and fat tissue were conducted using optical microscopy. **Results**: The HFD induced obesity in *Lgals3^+/+^* mice, but not in *Lgals3^−/−^* mice, which exhibited lower weight gain, food intake, daily energy intake, and energy efficiency than *Lgals3^+/+^* mice. Moreover, *Lgals3^−/−^* HFD mice had hyperglycaemia and hyperinsulinemia. Histological analysis revealed hypertrophied adipose tissue in *Lgals3^+/+^* HFD mice with abundant Gal-3^+^ crown-like structures, rarely observed in *Lgals3^−/−^* HFD mice. In the jejunum, *Lgals3^+/+^* HFD mice showed a significant reduction in Gal-3 expression in intestinal epithelial cells, whereas inflammatory signals were increased in *Lgals3^−/−^* HFD mice. In the liver, *Lgals3^+/+^* HFD mice showed significant steatosis and macrophages expressing Gal-3. In contrast, *Lgals3^−/−^* HFD mice showed pronounced hepatocyte ballooning, suggesting a more progressive stage of metabolic dysfunction-associated steatotic liver disease (MASLD). **Conclusions**: Together, these data suggest that Gal-3 protects the gut–liver axis and adipose tissue against cytotoxic effects caused by HFD.

## 1. Introduction

Galectin-3 (Gal-3) is a multifunctional β-galactoside-binding protein involved in several biological processes, including immunological responses, cellular interactions, and tumour progression [1]. Gal-3 is unique among the galectin family of proteins, as it is the only chimera-type galectin. It has an approximate molecular weight of 30 kDa, composed of a C-terminal carbohydrate recognition domain (CRD) and a non-lectin N-terminal domain that contains a unique proline–glycine–alanine–tyrosine-rich repeat motif. The roles of Gal-3 are diverse and depend on its cellular localization, including intracellular (cytoplasm and nucleus), extracellular (on the cell surface) or secreted into the extracellular environment [2].

The biological functions of Gal-3 have been associated with cellular events modulated by cell-to-cell adhesion and cell-to-extracellular matrix interactions [3]. Therefore, Gal-3 can potentially regulate fundamental cellular functions by affecting cell growth, specialization, and programmed cell death (apoptosis). During immunological responses and inflammation, Gal-3 regulates macrophage activation, leukocyte migration, and general inflammatory responses. It can promote either pro- or anti-inflammatory effects depending on the context [4,5]. Gal-3 has also been identified as a potential therapeutic target for tissue remodelling and fibrosis, serving as a key mediator in tissue repair and fibrogenesis [6,7].

During metabolic disruptions, Gal-3 has attracted attention due to its direct and indirect involvement in the pathogenesis of obesity, insulin resistance, and type 2 diabetes mellitus—T2DM. Gal-3 is commonly regarded as an essential connection between chronic low-level inflammation, a hallmark of obesity and type 2 diabetes mellitus (T2DM), and the metabolic issues that follow [8]. In this context, elevated circulating Gal-3 levels are strongly associated with insulin resistance in both obese subjects and mice [9]. Moreover, Gal-3 can promote the differentiation of pre-adipocytes into mature adipocytes (adipogenesis), suggesting a role in increasing fat mass [10]. Animal models can help to elucidate these questions.

The roles of Gal-3 have been studied in distinct experimental conditions [11,12]. Particularly in high-fat-diet (HFD) models, Gal-3 has emerged as a critical immunometabolic regulator, linking adipose tissue dysfunction to gut–liver axis impairment [13]. In adipose tissue, Gal-3 contributes to macrophage recruitment and activation, sustaining chronic low-grade inflammation [14]. In the gut–liver axis, Gal-3 plays protective roles in several conditions, including non-alcoholic fatty liver disease (NAFLD, now often referred to as MASLD—metabolic dysfunction-associated steatotic liver disease) and intestinal inflammation [15]. Collectively, these findings highlight Gal-3 as a multifunctional mediator in metabolic disease, indicating that it may represent a potential target for studies on diet-induced metabolic disorders.

Although previous studies have demonstrated important roles of Gal-3 in obesity, adipose tissue inflammation, and liver disease, its integrated contribution to the gut–adipose–liver axis during chronic high-fat-diet exposure remains poorly understood. In particular, the impact of Gal-3 deficiency on coordinated histopathological and inflammatory responses among these metabolically connected tissues has not been fully characterized. We therefore hypothesized that the absence of Gal-3 would exacerbate tissue dysfunction induced by an HFD along the gut–adipose–liver axis. Accordingly, this study aimed to investigate the effects of Gal-3 deficiency in mice chronically exposed to an HFD using Gal-3 knockout (*Lgals3*^−/−^) mice. The primary outcome was to evaluate histopathological alterations in the gut, visceral adipose tissue, and liver, while secondary outcomes included the assessment of inflammatory responses, adipose tissue remodelling, and intestinal and hepatic morphological changes associated with HFD-induced metabolic dysfunction. Together, these analyses were performed to further characterize the role of Gal-3 as a potential immunometabolic regulator involved in tissue responses to chronic dietary stress.

## 2. Materials and Methods

### 2.1. Animals

Male C57BL/6 mice aged 12 weeks, wild-type (WT; *Lgals3^+/+^*) and knockout for Gal-3 (KO; *Lgals3*^−/−^), from the Animal Facility of the Institute of Biomedical Sciences (ICB) at the Federal University of Rio de Janeiro, were housed under controlled temperature and humidity conditions (21 ± 2 °C, 50 ± 10%, respectively). Food and water were available *ad libitum*. The study received approval from an institutional animal care and use committee (IACUC), in this case, the Institutional Ethics Committee of the Federal University of Rio de Janeiro (Protocol number: 013/25).

### 2.2. High-Fat Diet

Mice were randomly divided into four groups and subjected to two diets throughout the experimental period: standard AIN93-M feed (74% carbohydrates, 14% protein, 12% lipids; energy content: 3800 kcal/g) or high-fat feed (26% carbohydrates, 14% protein, 60% lipids; energy content: 5400 kcal/g), both obtained from PragSoluções Biociências (São Paulo, Brazil). Table 1 provides information about the components of the diet. The four experimental groups were (I) CTR *Lgals3*^+/+^ Group: Wild-type animals fed standard feed (AIN93-M) containing 10% of calories in the form of lipids; (II) HFD *Lgals3*^+/+^ Group: Wild-type animals fed high-fat feed containing 60% of calories in the form of lipids; (III) CTR *Lgals3*^−/−^ Group: Gal-3 knockout animals fed a standard diet (AIN93-M) containing 10% of calories in the form of lipid; and (IV) HFD *Lgals3*^−/−^ Group: Gal-3 knockout animals fed an HFD containing 60% of calories in the form of lipid. All groups consisted of ten animals (*n* = 10 mice per group). The experimental animals received the respective diets for 90 days. Sample size was determined based on previous studies employing similar HFD-induced metabolic dysfunction models and considering ethical recommendations to minimize animal use.

### 2.3. Body Weight, Food Intake, Energy Efficiency, and Naso-Anal Length

On the first day of the experiment, researchers recorded the initial body weight before introducing the experimental diets. Animals were subsequently weighed every two weeks throughout the experimental period, totalling 90 days. Body weight gain was determined by subtracting the initial body weight from the final body weight during the 0–90-day treatment period. Body weight was determined using a Marte^®^ digital electronic balance, model ASF11 (Marte Cientifica, São Paulo, Brazil), offering a maximum capacity of 500 g and a precision of 0.01 g. Food intake was monitored daily by measuring the amount of diet provided to each cage. The remained food was weighed again on the following day. Thus, daily food consumption was calculated by subtracting the remaining diet from the initial amount of food. Energy efficiency was determined by calculating body weight gain per caloric intake. After the 90-day experiment concluded, researchers weighed the animals and determined the index for each group based on their total calorie consumption, using this formula:

Energy efficiency = (FBW − IBW)/kcal consumed

FBW represents the final body weight (g) at the end of the experimental period, IBW represents the initial body weight (g) at the beginning of the experiment, and kcal consumed corresponds to the total caloric intake during the analysed period. Once the animals were anesthetized and positioned on their backs, their naso-anal length was measured with a flexible tape marked in millimetres.

### 2.4. Fasting Blood Glucose

After weighing, every animal group underwent a fasting period lasting six hours. A drop of blood was collected from a 1 mm section at the extreme tip of the tail—far from the bone—to measure fasting blood glucose, which was subsequently analysed using a FreeStyle^®^ glucose meter (Abbott, São Paulo, Brazil). The fasting blood glucose levels of each animal were measured at the start of the experiment, as well as on days 15, 30, 60, 75, and 90.

### 2.5. Sample Collection

Immediately after euthanasia of the animals in a chamber with a controlled CO_2_ atmosphere, cardiac puncture was performed to take advantage of the last heartbeats. A 26G ½ needle syringe (insulin syringe) was inserted towards the right ventricle, and cardiac puncture was performed by suctioning the syringe to aspirate blood. A total of 1000 to 500 µL of blood was collected. The collected samples were centrifuged at 1200 rpm for 10 min at 4 °C to separate the serum, which was then stored at −20 °C until analysis.

### 2.6. Histological Analyses

For histological analyses, liver, visceral adipose tissue, small intestine, and large intestine samples were surgically collected, sectioned into ~1 mm^2^ fragments, and fixed in 4% neutral buffered formalin for 48 h. Following fixation, samples were washed three times in phosphate-buffered saline (PBS) for 5 min each to remove residual fixative. Tissues were dehydrated through a graded ethanol series (50% for 1 h, 70% for 1 h, and 100% ethanol in two steps of 30 min each). Subsequently, samples were cleared in xylene (two changes, 30 min each) and infiltrated with paraffin at 60 °C (two changes, 30 min each). After infiltration, tissues were embedded in paraffin blocks and sectioned at 3 µm thickness using a microtome (Leica Microsystems, Wetzlar, Germany). Sections were mounted on silanized slides (Knittel Glass, Braunschweig, Germany) and stored at room temperature until further use. For histological evaluation, sections were stained with haematoxylin and eosin (H&E) for general tissue morphology, periodic acid–Schiff (PAS) for assessment of glycogen content and distribution, and Gomori’s trichrome staining for visualization of collagen fibres and deposition. Brightfield images were acquired using an Axiophot microscope (Zeiss, Oberkochen, Germany) equipped with the AxioVision imaging system.

### 2.7. Immunohistochemistry

Silanized slides containing sections of liver, visceral adipose tissue, and small intestine embedded in paraffin were kept for twenty minutes in a dry oven at 60 °C. Then, the slides were subjected to the deparaffinization and rehydration process. After rehydration, antigen retrieval was performed, in which the slides containing the sections were placed in a beaker containing citrate buffer (pH 6.0) and subjected to a temperature of 90 °C in a steamer for 30 min. Then, the beaker was removed from the steamer and cooled to room temperature, and subsequently the slides were washed with distilled water to block endogenous tissue peroxidase. This procedure was performed by immersing the slides in a beaker containing 0.3% hydrogen peroxide diluted in methanol. The slides were then kept in this blocking for fifteen minutes and protected from light using aluminium foil. The slides were washed again in distilled water and subsequently washed three times with phosphate-buffered saline (PBS) + Tween 0.25% solution for five minutes each. Blocking of nonspecific epitopes was performed using PBS containing 10% bovine serum albumin (BSA) + 8% Molico milk (Nestlé, São Paulo, Brazil) on the sections to completely cover them, and these were kept for one hour at room temperature. After blocking, the slides were washed again three times with PBS + Tween 0.25% solution for five minutes each, and the sections were incubated at 4 °C in a humid chamber for 18 h (overnight) with primary antibody. After incubation with primary antibody, the slides were washed again three times with PBS + Tween 0.25% solution for 5 min each and the sections were incubated at room temperature in a humid chamber for 2 h with HRP or biotinylated secondary antibody.

After two hours of incubation with the secondary antibody, the slides were washed three times with PBS + Tween 0.25% solution for 5 min each time to reveal the staining with the DAB (diaminobenzidine) chromogen, which was diluted 1:50 in the kit’s own buffer and kept on the section until the peroxidase conjugated to the secondary antibody converted the DAB substrate into a brown precipitate. The length of time varied for each staining with different primary antibodies. Then, the reaction was stopped after immersing the slides in a beaker containing distilled water. After revelation with DAB, the sections were counterstained for 2 to 4 min (varying according to the tissue) with properly filtered Harris haematoxylin solution and subsequently washed in running water. For slide preparation, the sections were previously dehydrated in serial alcohol baths (95%, 100% I and 100% II) for five minutes each, followed by serial xylene baths (I, II and III) also for five minutes each, and the slides were covered with coverslips, using Entellan mounting medium. The sections were viewed and photographed using an Axiophot microscope with the AxioVision program (Zeiss, Oberkochen, Germany).

### 2.8. Insulin Quantification—Radioimmunoassay

Insulin levels were quantified from animal serum, using a competitive immunoradiological assay, using I125, as described in the kit (MP Biomedicals, Irvine, CA, USA). This technique evaluates the ability of the hormone in the samples to bind to specific antibodies in the presence of a hormone labelled with the radioactive element I125. Thus, it is a competitive assay, where analyte levels are inversely proportional to the radiation quantified by the ICN Isomedic 4/600 gamma particle counter (ICN Pharmaceuticals, Costa Mesa, CA, USA).

### 2.9. Morphometry of Adipocytes and CLSs Quantification

ImageJ version 1.54k (NIH, Bethesda, MD, USA) was utilised to carry out adipocyte morphometry. Histological sections of visceral adipose tissue from each experimental group were imaged using light microscopy at a consistent magnification of 100×. After calibrating with a reference scale, digital images were analysed to measure dimensions in micrometres. Adipocyte boundaries were identified by threshold-based segmentation followed by manual correction when necessary, and cells at the edges of the field or with incomplete membranes were excluded from analysis. For each sample, at least 100 adipocytes were quantified from randomly selected, non-overlapping fields. Cell area (µm^2^) was measured, and the equivalent diameter was calculated to account for variations in cell shape. To quantify crown-like structures (CLSs), complete rounded structures were identified and quantified per microscopic field at the same conditions to measure the adipocyte diameter. Data were expressed as mean values per animal and used for subsequent statistical analysis.

### 2.10. Morphometry of Intestinal Samples

The number of Gal-3+ cells in the intestine was quantified using ImageJ software (NIH, USA). All groups had histological images of both their small and large intestines taken under consistent conditions and assessed following scale calibration at 400× magnification. Positive cells were identified based on specific staining using colour thresholding (brown-stained cells), followed by manual verification to ensure accuracy. The total number of cells and the number of positively stained cells were counted in randomly selected, non-overlapping fields. Cells at the edges of the image were excluded. For each sample, 10 fields were analysed to minimize regional bias. The percentage of positive cells was calculated as the ratio of positive cells to total cells multiplied by 100, and the results were expressed as mean values per animal for statistical analysis.

### 2.11. Morphometry of Liver Samples

Hepatic steatosis was evaluated in histological sections using a semi-quantitative scoring system adapted from established criteria for non-alcoholic fatty liver disease (NAFLD). Briefly, sections were examined under light microscopy, and the extent of lipid accumulation was graded based on the percentage of hepatocytes containing lipid droplets: grade 0 (<5%), grade 1 (5–33%), grade 2 (34–66%), and grade 3 (>66%). Scoring was performed in multiple randomly selected, non-overlapping fields per sample by a blinded observer. In parallel, morphometric analysis was conducted using ImageJ (NIH, USA) to quantify the lipid area fraction after colour thresholding, allowing objective estimation of steatosis. Data were expressed as both histological scores and percentage of lipid area for statistical analysis.

Hepatocyte ballooning was assessed in liver sections using a semi-quantitative scoring system based on established histopathological criteria for steatohepatitis. Ballooned hepatocytes were identified by their enlarged size, rarefied cytoplasm, and loss of normal polygonal shape. The degree of ballooning was graded as follows: 0 (none), 1 (few ballooned hepatocytes), and 2 (many/prominent ballooned hepatocytes). Evaluation was performed in multiple randomly selected, non-overlapping fields per sample by a blinded observer. To support the analysis, morphometric assessment was conducted using ImageJ (NIH, USA), in which hepatocyte area and cytoplasmic characteristics were analysed after appropriate thresholding and manual correction. Data were expressed as ballooning scores per sample for subsequent statistical analysis.

### 2.12. Statistical Analysis

Sample size calculation was performed using G*Power software (G*Power 3.1.9.7. for Windows, Heinrich Heine University, Düsseldorf, Germany). The calculation was based on effect sizes reported in previous studies evaluating metabolic parameters in HFD-induced mice. Considering an alpha level of 0.05 and a statistical power of 80% (1 − β = 0.80), the minimum required sample size was estimated to be 10 mice per group. All statistical analyses were performed using GraphPad Prism software (version 11.0.2 (92)). Data are presented as mean ± standard error of the mean (SEM), and statistical significance was established at α = 0.05, with *p*-value ≤ 0.05 considered statistically significant. Data distribution was initially assessed using the Shapiro–Wilk normality test to justify the use of parametric analyses. Comparisons between two groups were performed using paired or unpaired two-tailed Student’s *t*-tests, as appropriate. For multiple comparisons, one-way or two-way ANOVA with repeated or non-repeated measures was applied according to the experimental design, followed by Tukey’s post hoc test for multiple-comparison correction when applicable. Outliers were evaluated by graphical inspection and were not excluded unless associated with clear experimental or technical inconsistencies. No missing data imputation was performed, and analyses were conducted using complete datasets obtained from the experimental groups. A complete statistical analysis is summarized in as Supplementary Material (Table S1).

## 3. Results

### 3.1. Gal-3 Deficiency Prevented Visceral Fat Accumulation and Weight Gain After Long-Time HFD

**Figure 1:**
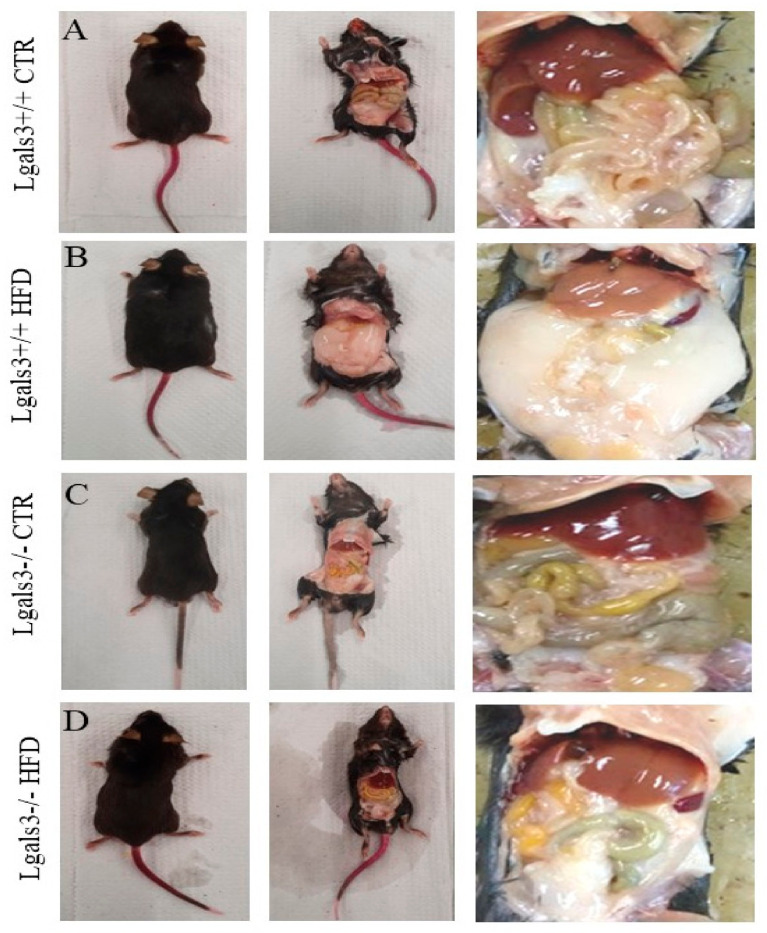
Effects of HFD and galectin-3 deficiency on body morphology and visceral adiposity. Macroscopic analysis of mice divided into four experimental groups: (**A**) *Lgals3^+/+^* control diet (CTR), (**B**) *Lgals3^+/+^* high-fat diet (HFD), (**C**) *Lgals3^−/−^* control diet (CTR), and (**D**) *Lgals3^−/−^* high-fat diet (HFD). Left panels show whole-body dorsal views, middle panels show ventral views after skin removal, and right panels display intra-abdominal organs and visceral adipose tissue (Amplification 10×). Data are representative of three independent experiments. *n* = 10 mice per group.

HFD intake is one of the primary experimental and physiological triggers for visceral adiposity and seems central to the development of insulin resistance and metabolic inflammation [16,17]. Macroscopic analysis indicated a robust accumulation of abdominal fat in HFD *Lgals3*^+/+^ mice when compared with control mice (Figure 1A,B). Moreover, these HFD *Lgals3*^+/+^ mice showed the liver extremely pale, indicating possible hepatic steatosis, i.e., fat accumulation in the liver in comparison with respective control groups (Figure 1A,B). In the absence of Gal-3, *Lgals3*^−/−^ mice fed a standard diet (CTR *Lgals3^−/−^*) presented rare visceral fat accumulation (Figure 1C). These Gal-3 deficient mice, however, did not show visible adiposity in the visceral fat tissue, significantly different from HFD *Lgals3*^+/+^ mice (Figure 1D). The absence of expanded visceral adipose tissue in *Lgals3*^−/−^ mice after HFD was linked to the liver with slightly pale coloration and signs of lobular retraction in some areas (Figure 1D). These data indicate that Gal-3 regulates adiposity dependent on HFD. On the other hand, its absence inhibited the expansion of visceral adipose tissue.

### 3.2. Gal-3 Interferes with Body Weight, Energy Intake, and Glucose Metabolism in HFD Murine Model

**Figure 2:**
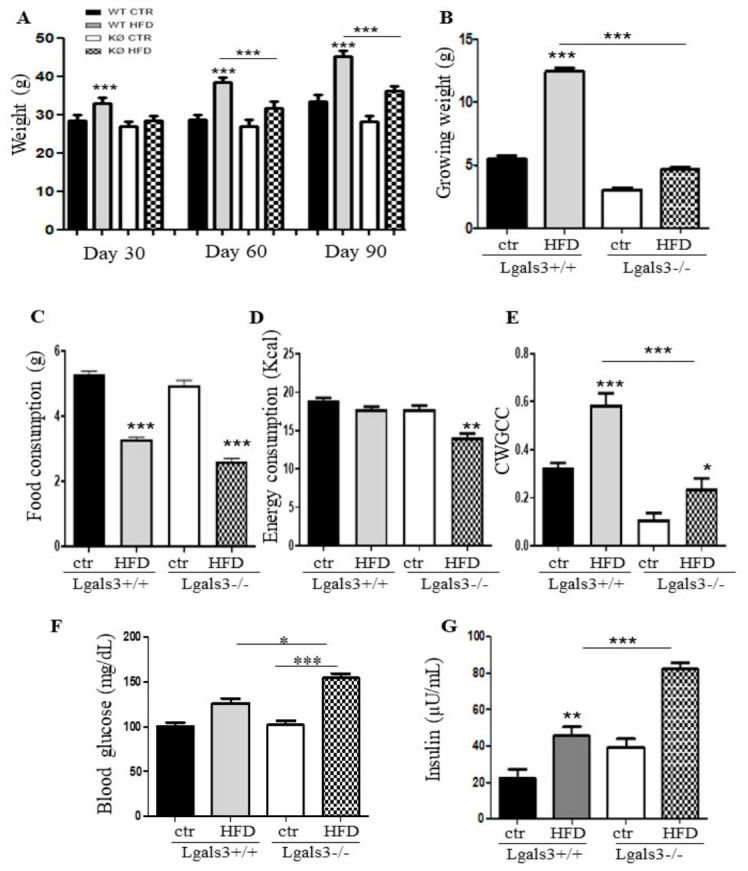
Impact of HFD and galectin-3 deficiency on body weight, metabolic parameters, and energy balance. (**A**) Body weight progression at days 30, 60, and 90 in wild-type (*Lgals3^+/+^*) and knockout (*Lgals3^−/−^*) mice fed the control diet (CTR) or HFD. (**B**) Total body weight gain over the experimental period. (**C**) Average daily food intake (g). (**D**) Energy intake (kcal). (**E**) Feed efficiency coefficient (GFCC), calculated as body weight gain relative to energy intake. (**F**) Fasting blood glucose levels (mg/dL). (**G**) Serum insulin levels (µU/mL). Data are representative of three independent experiments and are presented as mean ± SEM. *n* = 10 mice per group. Statistical significance is indicated as *p* < 0.05 (*), *p* < 0.01 (**), and *p* < 0.001 (***).

To better understand the phenotype of *Lgals3*^−/−^ mice fed an HFD, metabolic and physiological parameters were analysed in all experimental conditions. Exposure to an HFD significantly increased body weight gain in *Lgals3*^+/+^ wild-type animals compared with the other experimental groups. Among animals maintained on the control diet, *Lgals3*^+/+^ mice exhibited significantly greater weight gain than the *Lgals3*^−/−^ mice group, suggesting that the absence of Gal-3 negatively affects body weight gain even under basal conditions (Figure 2A). The *Lgals3^−/−^* group maintained on the control diet (*Lgals3*^−/−^ CTR) showed the lowest weight gain among all experimental groups (Figure 2A). Although the HFD also promoted weight gain in *Lgals3*^−/−^ HFD mice, this increase was significantly lower than that observed in *Lgals3*^+/+^ HFD mice, suggesting partial resistance to diet-induced weight gain in these animals (Figure 2B).

The analysis of food consumption revealed significant differences among the groups. Both the *Lgals3*^+/+^ HFD and *Lgals3*^−/−^ HFD groups showed a significant reduction in food intake (in grams) compared with animals maintained on the control diet (Figure 2C). These results indicate that food intake was primarily modulated by diet type, with reduced consumption in terms of food mass when animals were exposed to the HFD. When food intake was converted into energy consumption (kcal/day), the *Lgals3*^+/+^ CTR, *Lgals3*^+/+^ HFD, and *Lgals3*^−/−^ CTR groups showed relatively similar values of daily caloric consumption. However, *Lgals3*^−/−^ HFD mice exhibited a significant reduction in daily energy intake compared with the other experimental groups (Figure 2D), suggesting that, in addition to changes in food consumption by mass, the absence of Gal-3 may also impact energy balance under HFD conditions.

To evaluate the relationship between caloric intake and body weight gain, the coefficient of weight gain per caloric consumption (CWGCC), was calculated as an indicator of energy efficiency. The *Lgals3*^+/+^ HFD group showed the highest energy efficiency among all groups, with values significantly higher than those observed in the other groups (Figure 2E). This result indicates that, under an HFD, *Lgals3*^+/+^ mice convert ingested calories into body weight gain more efficiently. In contrast, *Lgals3*^−/−^ CTR animals showed the lowest energy efficiency coefficient, significantly lower than that of the other groups, indicating a lower capacity to convert ingested energy into body mass gain. Conversely, the *Lgals3*^−/−^ HFD group exhibited intermediate energy efficiency values, higher than *Lgals3*^−/−^ CTR but significantly lower than those observed in *Lgals3*^+/+^ HFD (Figure 2E). Together, these findings suggest that the absence of Gal-3 reduces the metabolic efficiency associated with HFD-induced weight gain.

To improve the metabolic screening, blood glucose and insulin levels were also measured in all experimental conditions. In *Lgals3*^+/+^ controls, the glucose levels were approximately 110 mg/dL. In *Lgals3*^+/+^ HFD mice, this value increased to 125 mg/dL. On the other hand, in the absence of Gal-3, glucose levels were around 100 mg/dL, but this value significantly increased to 150 mg/dL in the *Lgals3*^−/−^ HFD group (Figure 2F). In accordance, the insulin levels were compatible with glycemia. In *Lgals3*^+/+^ controls, the insulin concentration was near 20 µU/mL. In *Lgals3*^+/+^ HFD mice, this value increased to 40 µU/mL. In the absence of Gal-3, insulin levels were 30 µU/mL in *Lgals3^−/−^* CTR and 80 µU/mL in *Lgals3*^−/−^ HFD mice (Figure 2G), suggesting that the absence of Gal-3 can be associated with insulin resistance. In summary, the integrated analysis of these results indicated that an HFD induces a significant increase in body weight gain and energy efficiency in *Lgals3*^+/+^ mice. However, *Lgals3*^−/−^ mice exhibit an attenuated response to diet-induced weight gain. These findings suggest that Gal-3 may play an important role in the regulation of energy balance, susceptibility to diet-induced obesity, and insulin resistance in animal models of HFD.

### 3.3. The Absence of Gal-3 Modified the Adipose Tissue Organization in HFD Mice

**Figure 3:**
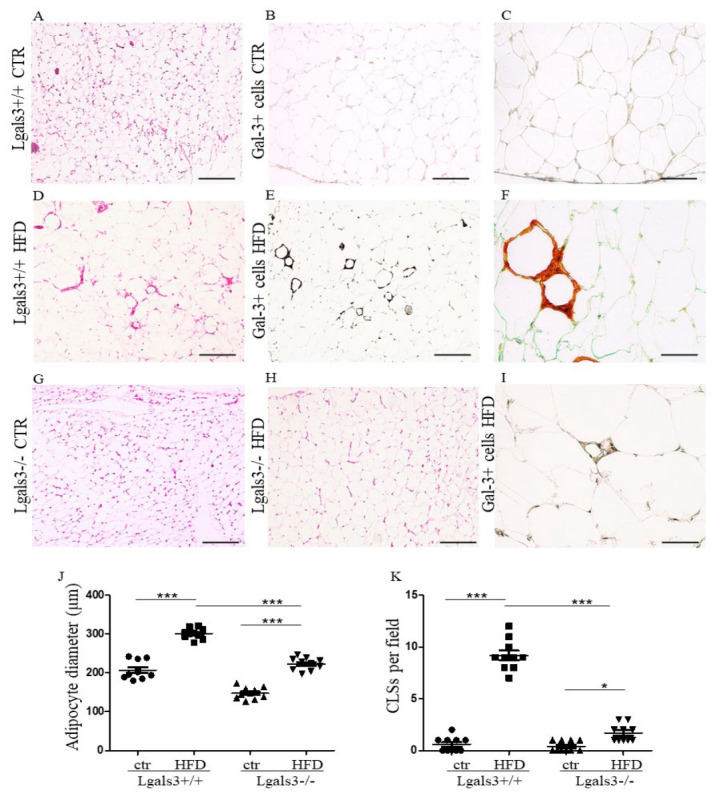
Histological analysis and galectin-3 distribution in visceral adipose tissue (VAT) of HFD-fed mice. Representative photomicrographs of VAT of mice. Samples of *Lgals3^+/+^* control diet (CTR) stained by haematoxylin and eosin (**A**) and immunohistochemistry to galectin-3 (**B**) with detailed image of VAT (**C**). Samples of *Lgals3^+/+^* high-fat diet (HFD) stained by haematoxylin and eosin (**D**) and immunohistochemistry to galectin-3 (**E**) with detailed image of VAT evidencing crown-like structures strongly positive to galectin-3 (**F**). Representative images of VAT from *Lgals3^−/−^* CTR diet (**G**) and *Lgals3^−/−^* HFD (**H**) both stained by haematoxylin and eosin. (**I**) Immunohistochemistry of galectin-3 on VAT of *Lgals3*^−/−^ HFD confirmed that these mice are negative to galectin-3. (**J**) Measurement of adipocyte diameter in micrometres. (**K**) Number of crown-like structures (CLSs) per microscopic field in amplification 100×. The data shown represent three separate experiments and are expressed as the mean ± SEM. *n* = 10 mice per group. Statistical significance is shown by *p* values: *p* < 0.05 (*), and *p* < 0.001 (***). Scale bars: 75 µm.

The subsequent experiments revealed that HFD feeding markedly altered visceral adipose tissue (VAT) morphology and inflammatory status in a Gal-3-dependent manner. In *Lgals3^+/+^* control samples, the VAT showed a typical histological structure with mostly unilocular adipocytes evenly spread throughout the tissue (Figure 3A). The expression of Gal-3 was scarce in these mice, although some adipocytes were palely stained in brown (Figure 3B,C). In parallel, the HFD diet induced a pronounced adipocyte hypertrophy in *Lgals3^+/+^* mice, hallmarked by enlarged lipid droplets and reduced cellular density. Moreover, several rounded well-stained structures were predominant in this HFD mice group, suggesting a crown-like structures (CLSs) formation (Figure 3D). Notably, Gal-3 showed clear labelling of the CLSs (see Figure 3E,F).

On the other hand, *Lgals3*^−/−^ mice displayed attenuated adipocyte enlargement and a preserved tissue architecture under the same dietary conditions (Figure 3G,H). As expected, samples of these mice were negative to Gal-3 and CLSs were rarely observed after fed an HFD (Figure 3I). Two quantitative data reinforced the differences in the adipocyte hypertrophy and CLS distribution in these experimental groups. The measurement of adipocyte perimeter confirmed a significant increase of adipocytes in HFD-fed wild-type animals (hypertrophy), whereas this effect was significantly blunted in Gal-3 knockout mice (Figure 3J). At the same time, *Lgals3*^+/+^ HFD showed a marked increase in macrophage infiltration forming CLSs, suggesting a heightened adipose tissue inflammation. In contrast, Gal-3 deficiency led to a significant reduction in immune cell accumulation (Figure 3K). Collectively, these findings indicate that Gal-3 plays a critical role in mediating adipocyte hypertrophy and inflammation in response to diet-induced metabolic stress.

### 3.4. Gal-3 Deficiency Disrupted the Mucosa of the Small Intestine After HFD

The accumulation of macrophages within adipose tissue is a central pathological process that drives adipose dysfunction and promotes metabolic disruptions associated with obesity. Overall, adipose tissue macrophages indicate local inflammation, which is closely linked to the gut–liver axis [18]. To investigate the involvement of Gal-3 in the obesity induced by HFD and the gut–liver axis, these organs were analysed by optical microscopy and immunohistochemical analysis.

**Figure 4:**
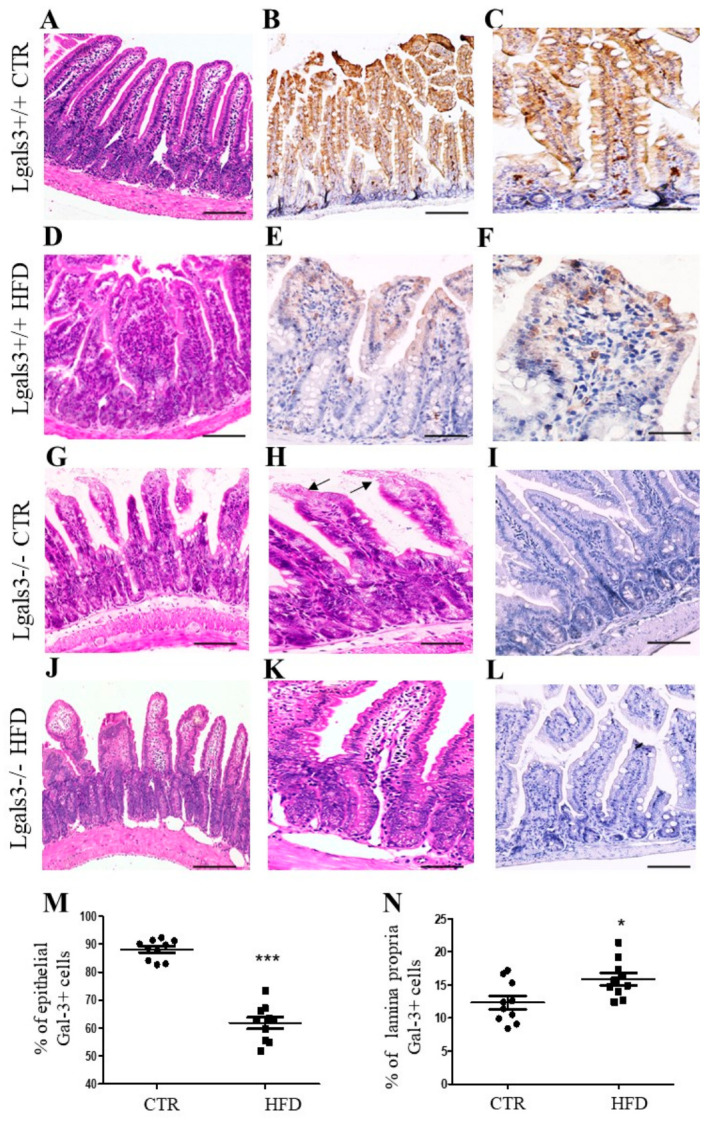
Histological analysis and galectin-3 expression in small intestine of HFD-fed mice. Representative images of intestinal sections showing mucosal architecture from *Lgals3^+/+^* CTR mice stained by haematoxylin and eosin (**A**) and galectin-3 ((**B**), immunohistochemistry). In detail, villi containing galectin-3 expressing cells ((**C**); brown signal). Samples of *Lgals3^+/+^* high-fat diet (HFD) stained by haematoxylin and eosin (**D**) and galectin-3 (**E**) with detailed image of jejunum evidencing rare epithelial cells expressing galectin-3 in the villus (**F**). Images of small intestine from *Lgals3^−/−^* CTR diet (**G**,**H**) show apparent disorganization of epithelial cells (arrows) and total absence of galectin-3 (**I**). In *Lgals3^−/−^* HFD mice, haematoxylin and eosin revealed oedematous villi (**J**,**K**) and confirmed that these mice are negative to galectin-3 (**L**). (**M**) Quantification of Gal-3-positive epithelial cells (in %). (**N**) Quantification of Gal-3-positive cells in the lamina propria (in %). Data are representative of three independent experiments and are presented as mean ± SEM. *n* = 10 mice per group. Statistical significance is indicated as *p* < 0.05 (*) and *p* < 0.001 (***). Scale bars: 150 µm.

In *Lgals3*^+/+^ CTR mice, haematoxylin and eosin staining of the jejunum revealed a well-preserved mucosal architecture (Figure 4A). In control mice, Gal-3 was clearly observed along the epithelial lining of the villi, showing intense staining within the cytoplasm (Figure 4B). Detailed analysis of villi confirmed that Gal-3 was strongly expressed by epithelial cells and elongated macrophage-like cells present in the lamina propria (Figure 4C). In contrast, the HFD modified the histological architecture of the jejunum.

*Lgals3*^+/+^ mice subjected to an HFD exhibited a moderate inflammatory response in the intestinal mucosa (Figure 4D). In addition, the distribution of Gal-3 in the gut mucosa of HFD mice was significantly altered in comparison to *Lgals3*^+/+^ CTR mice. These HFD-fed mice showed a noticeable reduction in Gal-3 immunoreactivity, particularly along the villus epithelium (Figure 4E). In more detail, it was possible to observe that most of the infiltrating leukocytes were mononuclear cells negative to Gal-3, except the macrophage-like cells (Figure 4F). Collectively, these findings indicate that an HFD disrupts the distribution of Gal-3 in epithelial cells of the intestinal mucosa.

In the absence of Gal-3, the intestinal mucosa of *Lgals3*^−/−^ CTR mice showed minimal disturbances when compared to wild-type controls (Figure 4G). Given that Gal-3 is strongly identified in the top of villi in *Lgals3*^+/+^ CTR mice, we analysed this region in samples obtained from *Lgals3*^−/−^ CTR mice. Although merely suggestive, some villi epithelial cells seemed significantly destroyed (Figure 4H, arrows). As expected, these samples were completely negative to Gal-3 (Figure 4I).

In the absence of Gal-3, the HFD caused more significant tissue damages. Samples of *Lgals3*^−/−^ HFD mice revealed a robust swelling in the lamina propria of the jejunum and lymphatic vasodilatation not only in the mucosa, but also in the submucosa layer (Figure 4J). In more detail, large lymphatic vessels were observed in the oedematous mucosa, frequently associated with leukocyte infiltration. In the submucosa, the high diameter of these vessels reinforced that *Lgals3*^−/−^ HFD mice accumulated tissue damage with probable interference in the lymph circulation (Figure 4K). Again, as expected, samples of *Lgals3*^−/−^ HFD mice were completely negative to Gal-3 (Figure 4L).

To quantify the impressive analysis of the Gal-3 distribution, epithelial and lamina propria cells expressing Gal-3 were measured in percentage terms in both *Lgals3*^+/+^ CTR and *Lgals3*^+/+^ HFD mice. In epithelial tissue of the gut mucosa, approximately 90% of cells in the villi were positive to Gal-3 in *Lgals3*^+/+^ CTR mice, whereas this percentage significantly decreased to around 60% of cells in *Lgals3*^+/+^ HFD mice (Figure 4M). In parallel, 12% of lamina propria cells expressed Gal-3 in *Lgals3*^+/+^ CTR mice, and this percentage increased to 17% in *Lgals3^+/+^* HFD mice (Figure 4N). Together, these data suggest that epithelial barrier can be severely affected by an HFD in a Gal-3 dependent manner.

### 3.5. Deficiency of Gal-3 Did Not Affect the Mucosa of the Large Intestine After HFD

**Figure 5:**
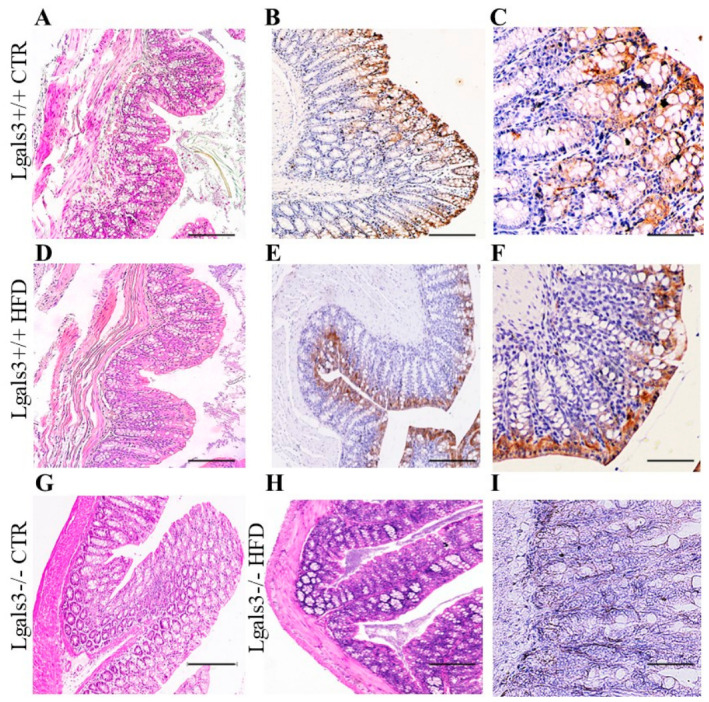
Colonic morphology and galectin-3 localization in HFD-fed mice. Representative images of colon sections from *Lgals3^+/+^* CTR mice stained by haematoxylin and eosin (**A**) and galectin-3 ((**B**), immunohistochemistry). In details, colonic mucosa showing cells positive to galectin-3 ((**C**); brown signal). Samples of *Lgals3^+/+^* high-fat diet (HFD) stained by haematoxylin and eosin (**D**) and galectin-3 (**E**) with amplified image of the colon containing epithelial cells expressing galectin-3 (**F**). Images of small intestine from *Lgals3^−/−^* CTR diet (**G**) and *Lgals3^−/−^* HFD mice (**H**) by haematoxylin and eosin staining. (**I**) sample of *Lgals3^−/−^* HFD mice completely negative to galectin-3. Data are representative of three independent experiments and are presented as mean ± SEM. *n* = 10 mice per group. Scale bars: 150 µm (**A**,**B**,**D**,**E**,**G**,**H**); 50 µm (**C**,**F**,**I**).

In the large intestine, Gal-3 distribution was monitored in all experimental groups. The histological architecture was well-characterized in *Lgals3*^+/+^ CTR mice. Mucosa containing crips and no villi, submucosa absent to inflammatory signals, and surrounding muscular layers were identified in these mice (Figure 5A). The majority of cells positive to Gal-3 were present in the epithelial barrier and partially observed in the crypts (Figure 5B,C). In contrast to the small intestine, the HFD did not disrupt the general histology of the large intestine. The colon of *Lgals3*^+/+^ HFD mice was similar to respective controls (Figure 5D).

The expression Gal-3 by epithelial cells was not modified in *Lgals3*^+/+^ mice fed an HFD. Although it was rare to observe Gal-3 expressing cells in the crypts, the distribution of this protein was similar in the epithelial barrier, in contact with the lumen, when compared to the *Lgals3*^+/+^ CTR mice (Figure 5E). In more detail, it was possible to reinforce that Gal-3 was well confined in the borderline of the mucosa (Figure 5F).

In *Lgals3*^−/−^ CTR mice, the colon showed no significant differences in comparison with *Lgals3*^+/+^ CTR mice (Figure 5G). On the other hand, the mucosa of the colon obtained from *Lgals3*^−/−^ HFD mice was hypercellularized in comparison with the respective controls. However, no organized leukocyte infiltration was found in these *Lgals3*^−/−^ HFD mice (Figure 5H). As expected, samples *Lgals3*^−/−^ CTR and *Lgals3*^−/−^ HFD mice (Figure 5I) were completely negative to Gal-3. Thus, the different distribution of Gal-3 in the small and large intestine after HFD suggested a potential regional influence in the epithelial barrier mediated by Gal-3.

### 3.6. Gal-3 Played Regulatory Roles in the Gut–Liver Axis in the HFD Mouse Model

The association between epithelial barrier disorders and hepatic damages was previously described in this experimental model [19]. In this context, intestinal disorders (especially in the epithelial barrier) are often associated with liver damage [20,21]. Moreover, hepatic Gal-3 was positively correlated with absence of steatosis and steatohepatitis in obese infants [22]. Then, the liver of all experimental groups was studied by optical microscopy and immunostaining to Gal-3.

**Figure 6:**
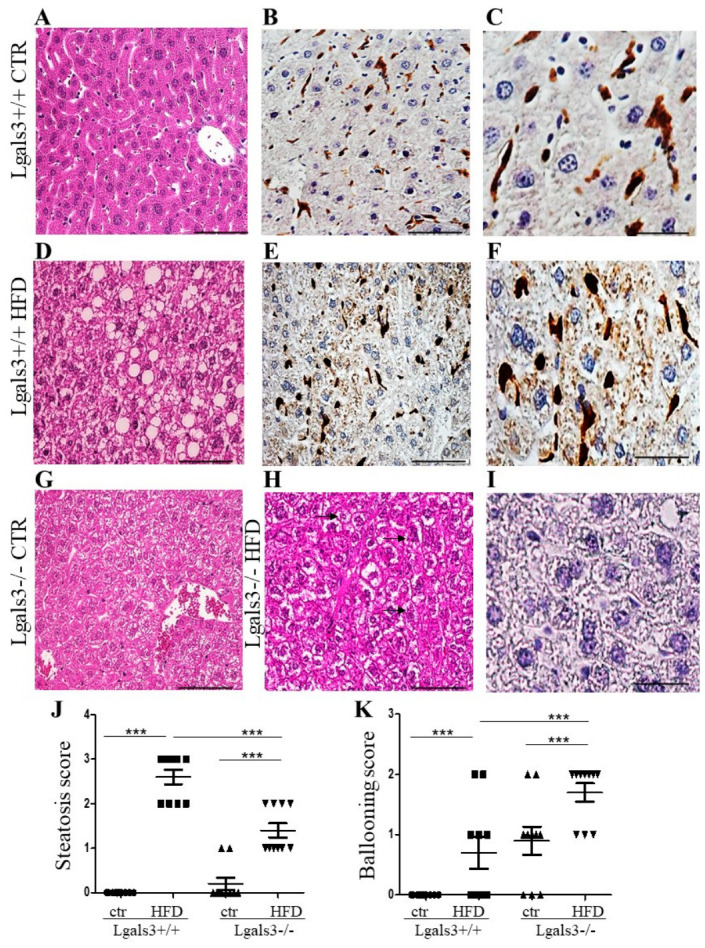
Histological analysis and galectin-3 expression in the liver of HFD-fed mice. Representative images of the liver from *Lgals3^+/+^* CTR mice stained by haematoxylin and eosin (**A**) and galectin-3 ((**B**), immunohistochemistry). In details, macrophages (Kupffer cells) strongly stained to galectin-3 ((**C**); brown signal). Samples of *Lgals3^+/+^* high-fat diet (HFD) stained by haematoxylin and eosin (**D**) and galectin-3 (**E**) with detailed image showing diffuse expression of galectin-3 by Kupffer cells and some hepatocytes (**F**). Images of the liver from *Lgals3^−/−^* CTR diet (**G**) and *Lgals3^−/−^* HFD mice (**H**) stained by H&E show apparent hepatocyte damages with Mallory–Denk bodies in *Lgals3^−/−^* HFD mice (**H**, arrows). Total absence of galectin-3 in these mice (**I**). (**J**) Steatosis score based on hepatic damages represented by lipid storage. (**K**) Ballooning score based on percentage of enlarged and paled hepatocytes. Data are representative of three independent experiments and are presented as mean ± SEM. *n* = 10 mice per group. Statistical significance is indicated as *p* < 0.001 (***). Scale bars: 25 µm (**A**,**B**,**D**,**E**,**G**,**H**); 75 µm (**C**,**F**,**I**).

In *Lgals3*^+/+^ CTR mice, the liver showed a classic organization. Hepatocytes were well preserved in the parenchyma, intercalated by sinusoids containing Kupffer cells (Figure 6A). The immunohistochemical analysis showed that the Kupffer cells were significantly immunolabelled to Gal-3, whereas hepatocytes were negative (Figure 6B). In detail, Gal-3 was detected in elongated cells within the sinusoids, reinforcing that the Kupffer cells were positive to Gal-3 (Figure 6C).

The HFD induced a severe liver steatosis in *Lgals3*^+/+^ HFD mice group. Macro- and microvesicles of lipids were frequently observed in several hepatocytes. Furthermore, part of these cells showed ballooning morphology, suggestive of progressive steatosis to steatohepatitis (Figure 6D). Kupffer cells maintained the expression of Gal-3, but some parenchymal cells were also stained to Gal-3 (Figure 6E), although it was difficult to prove the specificity of the reaction, because most of the hepatocytes was not intact. In detail, the strong immunoreaction to Gal-3 was observed in Kupffer cells. However, a pigmented staining to Gal-3 was detected in part of the hepatocytes (Figure 6F).

In the absence of Gal-3, liver samples revealed important histological disruptions. In *Lgals3^−/−^* CTR mice, some enlarged hepatocytes with a rounded shape and pale, rarefied cytoplasm, and apparent cytoskeletal disruption were observed (Figure 6G). The HFD amplified this phenotype, and clearly, these *Lgals3^−/−^* HFD mice showed a severe hepatocyte ballooning (Figure 6H). Hepatocyte ballooning was identified by cytoplasmic inclusions known as Mallory–Denk bodies (Figure 6H, arrows). As expected, these samples were completely negative to Gal-3 (Figure 6I).

Morphometric and histopathological parameters revealed that *Lgals3*^+/+^ HFD showed relevant steatosis scores. In numbers, 60% of these mice showed steatosis grade 3 (score maximum) and 40% grade 2, while all *Lgals3*^+/+^ CTR were negative to steatosis (Figure 6J). On the other hand, *Lgals3*^−/−^ HFD mice were divided into 60% with steatosis grade 2 and 40% grade 1 (Figure 6J). Notably, 20% of *Lgals3*^−/−^ CTR mice showed steatosis grade 1 (Figure 6J). However, histological images of *Lgals3*^−/−^ HFD mice demonstrated more progressive damage in the liver. Only 20% of *Lgals3*^+/+^ HFD mice showed hepatocyte ballooning in grade 2 (score maximum), whereas 70% of *Lgals3*^−/−^ HFD mice developed to this stage (Figure 6K). Indeed, 50% of *Lgals3*^+/+^ HFD mice were negative to hepatocyte ballooning, while all *Lgals3*^−/−^ HFD mice showed this progressive disease marker (Figure 6K). These findings indicate that Gal-3 plays hepatoprotective functions, given that in the absence of Gal-3, significant hepatocellular injury and critical hallmarks of progressive liver disease were observed.

## 4. Discussion

Gal-3 has been associated with several metabolic diseases. Although the underlying mechanisms remain unclear, this manuscript reveals an important contribution of Gal-3 to progressive disorders in the gut–liver–adipose axis. Here, it was demonstrated that *Lgals3*^−/−^ mice subjected to an HFD did not develop obesity, but they presented critical disturbances in the intestinal mucosa, liver parenchyma, and adipose tissue biology. In general, these results indicate that Gal-3 plays protective roles in the gut–liver axis and positively regulates adipose tissue inflammation.

The HFD induced obesity only in *Lgals3*^+/+^ mice. In contrast, *Lgals3*^−/−^ mice fed an HFD showed only a modest weight gain. The differences observed in food intake can be relevant to validate the experimental model. As expected, the HFD induced a significant reduction in food consumption in both *Lgals3*^+/+^ and *Lgals3*^−/−^ supplemented mice. Indeed, this experimental diet induces obesity in C57BL/6 mice [23]. However, why *Lgals3*^−/−^ mice did not develop obesity remains unclear.

Considering the differences observed in energy intake among the experimental groups, we further addressed this issue by analysing energy efficiency, calculated as body weight gain relative to total caloric intake. The CWGCC used as an indicator of energy efficiency suggested that the *Lgals3*^+/+^ HFD group showed the highest energy efficiency among all other experimental groups. These findings suggest that the attenuated weight gain observed in Gal-3-deficient mice was not solely explained by reduced caloric intake but also reflected differences in the efficiency of converting ingested energy into body mass. This interpretation is supported by previous studies demonstrating that susceptibility to diet-induced obesity is not exclusively determined by caloric intake, but also by differences in metabolic and feed efficiency, energy utilization, and genetic background [24]. Therefore, the reduced weight gain observed in *Lgals3*^−/−^ HFD mice likely reflects not only lower energy intake but also impaired efficiency in converting ingested calories into body mass. Perhaps, these mice were able to convert ingested calories into body weight efficiently. Mechanisms affected by the absence of Gal-3 remain unclear, although it is important to note that *Lgals3*^−/−^ CTR mice showed the lowest energy efficiency coefficient in comparison with other groups. Thus, it is plausible to suggest that in deficiency of Gal-3, mice can have a lower capacity to convert ingested energy into body mass gain.

HFDs commonly induce hyperglycaemia and insulin resistance in mice, serving as a standard model for studying type 2 diabetes. Experimental models using HFDs over prolonged time periods (6–12 weeks) are hallmarked by elevated glycemia, higher plasma insulin, reduced glucose clearance, and increased body weight [24,25,26]. In accordance, we observed that our HFD induced a significant increase of glycemia and insulinemia in both *Lgals3*^+/+^ and *Lgals3*^−/−^ mice. However, the glucose and insulin levels were higher in the absence of Gal-3 than in wild-type mice. In terms of glucose metabolism and accumulation in *Lgals3*^−/−^, previous data corroborate our findings. Pugliese and colleagues demonstrated that *Lgals3^−/−^* mice developed severe diabetic glomerulopathy [27]. Darrow and colleagues revealed that Gal-3 deficiency aggravates hyperglycaemia and other diabetes-related complications [28]. Together, these findings suggest that Gal-3 may play an important role in the regulation of energy balance, glucose metabolism and susceptibility to diet-induced obesity.

In HFD mouse models, adipose tissue typically exhibits marked hypertrophy of adipocytes, increased macrophage infiltration, and chronic low-grade inflammation. These morphological aspects reflect progressive tissue dysfunction [29]. By macroscopic analysis, we observed that wild-type *Lgals3*^+/+^ HFD mice developed obesity associated with an increased region of visceral adipose mass. On the other hand, the visceral adipose tissue of *Lgals3*^−/−^ HFD mice did not expand after feeding with an HFD. To try to understand these phenotypes, we investigated other parameters. Several morphological differences were described here, including significant structural changes in the gut–liver axis and visceral adipose tissue in HFD-fed mice. In general, the absence of Gal-3 revealed important targets in the tissues involved with the HFD experimental model.

In *Lgals3*^+/+^ HFD mice, most adipocytes were enlarged, typically suggesting hypertrophy. On the other hand, *Lgals3*^−/−^ HFD mice did not develop adipocyte hypertrophy in the visceral adipose tissue. One possible association can be attributed to CLS formation. CLSs are macrophages which often accumulate around dead adipocytes, caused by excess nutrient intake, forming the characteristic “crown-like” morphology. These events trigger monocyte recruitment that differentiates into pro-inflammatory macrophages which are particularly associated with chronic low-grade inflammation, increased secretion of cytokines, and enhanced lipolysis [30,31]. More recently, these cells were also correlated with metabolic disturbances [32].

Gal-3 seems critical to CLS biology, considering that CLSs were significantly increased in *Lgals3*^+/+^ HFD mice, strongly positive to Gal-3 in these mice, and rarely observed in *Lgals3*^−/−^ HFD mice. These findings reinforce the suggestive association between Gal-3 and inflammatory responses in the adipose tissue [33]. In accordance, Gal-3 promotes chronic low-grade inflammation through macrophage activation and cytokine release [34], possibly contributing to adipose tissue dysfunction and systemic insulin resistance.

Recently, it was demonstrated that Gal-3 levels are increased in the bloodstream after 16 weeks of HFD [35]. However, the roles of Gal-3 in HFD models seem ambiguous in the literature. In accordance with our findings, Baek and colleagues demonstrated that *Lgals3*^−/−^ mice had lower body weight, adipose tissue mass and lipogenic gene expression than *Lgals3^+/+^* mice after a 12-week HFD [10]. Corroborating those findings, Fantauzzi and colleagues demonstrated that *Lgal3*^−/−^ mice showed low adipocyte size and expression of adipogenic genes, resulting in impaired adipose tissue maturation [14]. In contrast, Pang and colleagues demonstrated that *Lgals3*^−/−^ mice developed excessive adiposity and systemic inflammation after 24 weeks of diet-induced obesity [36]. In accordance with them, Jeftic and colleagues revealed that *Lgals3*^−/−^ mice develop more severe hepatic steatosis and increased visceral adiposity than *Lgals3*^+/+^ mice after a 24-week HFD [15]. These contradictory finings remain unexplained, although both protocols were different. Here, we used the protocol of Baek and collaborators. Consistently, low body weight and adiposity in the absence of Gal-3 were also observed in our work.

Regarding glucose homeostasis, there are fewer conflicts in the literature. Darrow and Shohet showed that *Lgals3*^−/−^ HFD mice had greater hyperglycemia and impaired glucose tolerance than *Lgals3*^+/+^ HFD mice after 8 weeks of the diet [28]. Consistently, *Lgals3*^−/−^ mice induced to diabetes with streptozotocin developed accelerated glomerulopathy 4 months later when compared with *Lgals3*^+/+^ mice [27]. Here, we demonstrated that glucose and insulin levels significantly increased in *Lgals3*^−/−^ HFD-fed mice in comparison with *Lgals3*^+/+^ HFD mice.

It is important to note that the HFD model classically induces adiposity and obesity [37]. However, the absence or reduction of adiposity does not necessarily confer metabolic protection. In contrast, it can instead exacerbate dysfunction along the gut–liver axis. Thus, HFD-induced liver injury can occur independently of obesity, as occurs in non-alcoholic fatty liver disease (NAFLD) [38]. Given that *Lgals3*^−/−^ HFD mice did not develop obesity, we decided to investigate the involvement of Gal-3 in orchestrating associated functions in related tissues, including the gut and liver.

HFD-associated alterations in the gut–liver axis, such as increased intestinal permeability and endotoxin translocation, further exacerbate hepatic injury [39,40]. These processes contribute to histopathological features including hepatocyte ballooning and inflammatory infiltration even in the absence of significant weight gain. Indeed, these hepatic damages can be directly associated with atypical glycogenosis and metabolic reprogramming [41].

In the gut, HFDs promote structural and functional disruptions that contribute to systemic metabolic disease. HFD feeding disrupts gut barrier integrity by reducing tight junction proteins (e.g., occludin, claudins, zonulins), leading to increased intestinal permeability [22]. In parallel, Gal-3 directly impacts the stability of tight junctions, including zonulin-1, occludin, and claudins [42]. Thus, it is plausible to suggest that Gal-3 also regulates cell–cell interactions in the epithelial tissue of intestinal mucosa. In this context, samples of *Lgals3*^+/+^ mice showed an intense staining to Gal-3 by intestinal epithelial cells in the luminal barrier of controls. However, there was a significant reduction in the positivity to Gal-3 after being fed an HFD. Recently, using a model of a gluten-rich diet, we also detected a robust reduction in the expression of Gal-3 by intestinal epithelial cells [43]. In both experimental conditions, important histological changes were observed in the gut.

It was previously described that intestinal epithelial cells of *Lgals3*^−/−^ mice had important alterations in the barrier function. Enterocytes of *Lgals3^−/−^* mice exhibited atypical cytoarchitecture resulting in protrusions in the basolateral membranes, intracellular vesicles and vacuoles, and aberrant distribution of villi [44]. Furthermore, the guts of *Lgals3*^−/−^ mice also showed impaired epithelial cell migration and delayed wound healing [45], reduced ability to maintain tight junction organization [44], and increased susceptibility to epithelial damage. Together, it is plausible to suggest that reduction of Gal-3 after HFD can be associated with barrier dysfunctions.

The HFD model can be very useful to investigate the interference of Gal-3 in the gut–liver axis. In metabolic settings (e.g., HFD), these epithelial defects contribute to worsened barrier dysfunction and can potentiate systemic effects such as endotoxemia and progression of NAFLD [46,47]. Functionally, Gal-3 deficiency is frequently associated with increased intestinal permeability, allowing greater translocation of luminal antigens and bacterial products affecting the liver [42]. In the context of an HFD, Gal-3 seems to play a central and bidirectional role in the gut–liver axis, integrating intestinal barrier function, immune activation, and hepatic metabolic responses.

Once in the liver, microbially derived signals trigger Kupffer cell activation and hepatocellular stress [48]. In our experimental conditions, we confirmed that Gal-3 is also highly expressed by Kupffer cells contributing to immunological aspects in the liver. In this way, we have considerable evidence to suggest that Gal-3 acts as a key mediator linking gut-derived endotoxemia to hepatic immune and metabolic dysfunction. Although the livers of both *Lgals3*^+/+^ and *Lgals3*^−/−^ HFD mice did not develop fibrosis, these mice responded differently to the HFD. The liver of *Lgals3*^+/+^ HFD mice was marked by steatosis whereas *Lgals3*^−/−^ HFD mice developed hepatocyte ballooning typically present in steatohepatitis. Thus, Gal-3 can be considered a critical regulator of HFD-induced gut–liver axis alterations, influencing the development and progression of NAFLD through coordinated effects on intestinal permeability, immune signalling, and hepatic injury.

Liver steatosis is hallmarked by accumulation of triglycerides within hepatocytes, and it was included as *sine qua non* condition to development of NAFLD [49]. Recently, NAFLD was formerly renamed to metabolic dysfunction-associated steatotic liver disease (MASLD), maintaining the major histopathological characteristics, such as excessive lipids in the liver, exceeding 5% of liver weight [50]. Progressive MASLD is hallmarked by hepatocyte ballooning, posteriorly to the steatosis stage. These morphological changes in the hepatocytes are associated with cellular injury and structural degeneration of the tissue [51]. Given that we observed significant hepatocyte ballooning in samples of *Lgals3*^−/−^ HFD mice and typical steatosis in *Lgals3*^+/+^ HFD mice, our data suggest that Gal-3 plays protective roles in the liver in the HFD model.

Morphological parameters reinforced this premise, considering that samples with liver steatosis show clear cytoplasmic lipid vacuoles, nucleus typically displaced and cell architecture preserved. On the other hand, hepatocyte ballooning is a key criterion distinguishing simple steatosis from non-alcoholic steatohepatitis, strongly associated with disease progression. It is characterized by frequent enlarged (swollen) hepatocytes with pale, rarefied, “flocculent” cytoplasm. Moreover, these cells are pathologically identified by Mallory–Denk bodies indicating cytoskeleton degeneration [52,53]. Hepatocyte ballooning is therefore considered a critical indicator of active liver injury and worse prognosis. Comparing *Lgals3*^+/+^ with *Lgals3*^−/−^ HFD mice, all these histological features were respectively detected in each mice group, reinforcing the possible protective roles of Gal-3.

While steatosis is often reversible and not necessarily associated with injury, ballooning is directly associated with oxidative stress, lipotoxicity, endoplasmic reticulum stress and mitochondrial dysfunction, indicating active hepatocellular damage [54]. In this context, hepatic steatosis and progression to cellular injury are driven less by total adiposity and more by metabolic dysfunction, including ectopic lipid accumulation, adipose tissue dysregulation, and altered lipid flux to the liver. Moreover, dysfunctional adipose tissue may fail to adequately buffer circulating free fatty acids, promoting hepatic lipotoxicity and inflammation [55]. These arguments reinforce the suggested protective role of Gal-3 in our experimental model.

The necessity of obesity to develop MASLD is controversial, although the major complications described are really linked to obesity. Phipps and Wattacheril suggested that MASLD in non-obese individuals would be attributed to predisposed genetic polymorphisms associated with insulin resistance, atherogenic dyslipidaemia and alterations in body composition [56]. In this context, Tan and colleagues demonstrated that a significant proportion of non-obese NAFLD (MASLD) patients have NASH or advanced fibrosis in comparison with non-obese NAFLD patients with advanced liver disease [57]. Indeed, there is robust evidence that beneficial effects on MASLD may not necessarily require weight loss [58]. Interestingly, although obesity-related alterations were more pronounced in *Lgals-3*^+/+^ HFD-fed mice, MASLD severity appeared comparatively milder than expected in these mice when compared with *Lgals3^−/−^* HFD-fed mice. These data point to Gal-3 as a potential molecular target for future studies showing similar phenotypes.

This study has some limitations that should be acknowledged underlying the effects of Gal-3 deficiency. First, it primarily focused on histopathological alterations and did not include extensive molecular analyses to further elucidate possible mechanistic pathways. Another limitation is the exclusive use of a murine model, which may restrict direct translation of the findings to human metabolic diseases. Importantly, although the HFD model allowed the investigation of diet-induced metabolic dysfunction under physiologically relevant conditions, differences in energy intake between groups may have influenced some metabolic and inflammatory outcomes. Future studies should incorporate pair-feeding strategies, larger experimental cohorts, and complementary molecular and functional analyses to better define the immunometabolic mechanisms linking Gal-3 to gut–adipose–liver-axis dysfunction during chronic HFD exposure.

## 5. Conclusions

In conclusion, Gal-3 emerges as a critical regulator of inter-organ interactions in the gut–liver axis. Our findings challenge the prevailing paradigm that obesity is a prerequisite for diet-induced liver injury by demonstrating that HFD exposure alone is sufficient to induce gut–liver axis dysfunction independently of adipose tissue expansion. This dissociation repositions the gut–liver axis, rather than adiposity per se, as a central driver of metabolic pathology. These results redefine the spectrum of MASLD by supporting the existence of a non-obese phenotype driven by diet-induced alterations in gut and liver homeostasis. Further studies are required to delineate the underlying molecular mechanisms and translate these findings to human settings. This work establishes a new conceptual framework and highlights the gut–liver axis including Gal-3 as a promising target for therapeutic intervention beyond the traditional focus on obesity.

## Figures and Tables

**Table 1:** Diet composition, energy content, and percentage of macronutrients (PragSoluções Biociências (São Paulo, Brazil).

Ingredients	Standard Diet (AIN93-M)	High-Fat Diet
Casein (g)	140.0	180.1
Corn starch (g)	465.7	147.6
Dextrinized starch (g)	155.0	155.0
Sucrose (g)	100.0	100.0
Soybean oil (g)	40.0	40.0
Lard (g)	-----	278.0
Fiber (g)	50.0	50.0
Mineral mix (g)	35.0	35.0
Vitamin mix (g)	10.0	10.0
L-cysteine (g)	1.8	1.8
Choline bitartrate (g)	2.5	2.5
Antioxidants (g)	0.008	0.008
Energy (kcal)	3800	5400
**Percentage of macronutrients** **(% kcal)**		
Carbohydrates (%)	74	26
Proteins (%)	14	14
Lipids (%)	12	60

Dietary composition and energy content per 1000 g (1 kg).

## Data Availability

The data used to support the findings of this study are available from the corresponding author upon request.

## Supplementary Materials

The following supporting information can be downloaded at: https://www.mdpi.com/article/10.3390/biomedicines14061288/s1, Table S1: Supplementary material to appropriate statistical analysis.
