# Supplementary material for: Lack of Galectin-3 Disturbs Gut–Adipose–Liver Axis in High-Fat-Diet Mice Model
Source: Biomedicines. 2026 Jun 5;14(6):1288. doi: 10.3390/biomedicines14061288 (PMC13297068; doi:10.3390/biomedicines14061288)
Supplement: Supplementary file 1 [file biomedicines-14-01288-s001.zip › biomedicines-4319850-supplementary.pdf]

Table S1: Supplementary material to appropriate statistical analysis.

**Parameter**	**WT-CTR**	**WT-HFD**	**KO-CTR**	**KO-HFD**	**Overall p**	**Significant post hoc comparisons**
Body weight (30 days)	28.2 ± 1.4	36.3 ± 1.7	27.5 ± 1.6	28.7 ± 1.5	0.9817	Not applicable
Body weight (60 days)	28.6 ± 1.3	40.2 ± 0.9	28.8 ± 3.5	30.4 ± 1.3	0.0002	WT CTR vs WT HFD<br>WT HFD vs KO CTR<br>WT HFD vs KO HFD<br>KO CTR vs KO HFD
Body weight (90 days)	33.1 ± 3.9	46.7 ± 2.8	29.7 ± 2.9	35.4 ± 3.9	< 0.0001	WT CTR vs WT HFD<br>WT HFD vs KO CTR<br>WT HFD vs KO HFD<br>KO CTR vs KO HFD
Weight gain	5.5 ± 1.4	12.4 ± 1.7	3.1 ± 1.6	4.7 ± 1.5	0.2512	not applicable
Food consumption	5.1 ± 0.6	3.5 ± 0.4	4.7 ± 0.9	2.9 ± 0.4	< 0.0001	WT CTR vs WT HFD<br>WT CTR vs KO HFD<br>WT HFD vs KO CTR<br>KO CTR vs KO HFD
Energy consumption (Kcal)	18.5 ± 1.5	16.4 ± 1.4	17.1 ± 1.1	13.9 ± 1.4	< 0.0001	WT CTR vs WT HFD<br>WT CTR vs KO HFD<br>WT HFD vs KO HFD<br>KO CTR vs KO HFD
Coefficient of weight gain per caloric consumption	0.35 ± 0.05	0.62 ± 0.07	0.19 ± 0.05	0.23 ± 0.03	< 0.0001	WT CTR vs WT HFD<br>WT CTR vs KO CTR<br>WT CTR vs KO HFD<br>WT HFD vs KO CTR<br>WT HFD vs KO HFD
Blood glucose (mg/dL)	101.1 ± 4.7	123.2 ± 10.8	102.2 ± 12.8	144.1 ± 8.7	< 0.0001	WT CTR vs WT HFD<br>WT CTR vs KO HFD<br>WT HFD vs KO CTR<br>WT HFD vs KO HFD<br>KO CTR vs KO HFD
Insulin (µU/mL)	19.8 ± 2.1	41.1 ± 3.5	33.1 ± 2.7	77.4 ± 4.1	< 0.0001	WT CTR vs WT HFD<br>WT CTR vs KO CTR<br>WT CTR vs KO HFD<br>WT HFD vs KO CTR<br>WT HFD vs KO HFD<br>KO CTR vs KO HFD

Planned comparisons between specific experimental groups (CTR vs. HFD and Lgals3+/+ (WT) vs. Lgals3-/- (KO)) were performed based on a priori biological hypotheses.

Significant after T-Test ($p<0.05$ in planned comparisons):

Body weight – 30 days: WT CTR vs WT HFD; WT HFD vs KO HFD

Weight gain: WT CTR vs WT HFD; WT HFD vs KO HFD; KO CTR vs KO HFD
